# A telehealth approach to improving clinical trial access for infants with tuberous sclerosis complex

**DOI:** 10.1186/s11689-019-9302-0

**Published:** 2020-01-22

**Authors:** Carly Hyde, Maria Pizzano, Nicole M. McDonald, Charles A. Nelson, Connie Kasari, Elizabeth A. Thiele, Shafali S. Jeste

**Affiliations:** 1 UCLA Semel Institute for Neuroscience and Human Behavior, Los Angeles, CA 90095 USA; 2 UCLA Graduate School of Education and Information Studies, Los Angeles, CA 90095 USA; 3 UCLA Semel Institute, Los Angeles, CA 90095 USA; 4 Boston Children’s Hospital and Harvard Medical School, Harvard Graduate School of Education, Boston, MA 02115 USA; 5 Massachusetts General Hospital and Harvard Medical School, Herscot Center for Tuberous Sclerosis Complex, Boston, MA 02114 USA

**Keywords:** Telehealth, Early intervention, Tuberous sclerosis complex, Clinical trial recruitment, Behavioral intervention, Autism spectrum disorder, Remote delivery

## Abstract

**Background:**

Research in rare genetic syndromes associated with ASD is often hampered by the wide geographic distribution of families and the presence of medical comorbidities, such as epilepsy, that may preclude travel to clinical sites. These challenges can limit the sample size and generalizability of the cohorts included in both natural history studies and clinical trials. Tuberous sclerosis complex (TSC) is a rare genetic syndrome that confers an elevated risk for autism spectrum disorder (ASD), with social communication delays identified in this population as early as 12 months of age. Early identification of risk necessitates parallel testing of early intervention, prompting the first randomized controlled clinical trial of behavioral intervention for infants with TSC (NCT03422367). However, considerable early recruitment challenges have mandated the systematic identification of enrollment barriers followed by modification of the study design to address these barriers.

**Methods:**

Caregivers were interviewed regarding barriers to enrollment (phase 1). Adaptations to the intervention were made to address these barriers (phase 2). Outcomes based on this modification to the study design were defined by enrollment rate and participant demographics.

**Results:**

Qualitative reports from caregivers indicated that distance and time were the primary barriers to clinical trial enrollment. The intervention was then modified to a remote model, with at-home, parent-delivered intervention, and weekly video conferencing with interventionists at the study sites. Enrollment increased 10-fold (from 3 to 30 participants) within 1 year and included a more diverse and clinically representative cohort of infants.

**Conclusion:**

The design and implementation of more scalable methods to disseminate research remotely can substantially improve access to clinical trials in rare neurodevelopmental disorders. The lessons learned from this trial can serve as a model for future studies not only in rare conditions, but in other populations that lack adequate access, such as families with limited financial or clinical resources. Continued efforts will further refine delivery methods to enhance efficiency and ease of these delivery systems for families.

## Background

Over the last decade, a host of identified genetic etiologies associated with autism spectrum disorder (ASD) have helped to disentangle the vast heterogeneity of the condition and inform more targeted treatments. Each of these genetic syndromes is rare (with prevalence rates ranging from 1/50,000 to 1/6000), but together they account for up to 15% of ASD [1]. Children with “syndromic” autism share common clinical features, including varying degrees of co-occurring conditions such as intellectual disability, motor impairment, epilepsy, sleep problems, and gastrointestinal dysfunction, necessitating timely treatments. Many of these syndromes manifest in infancy, with epilepsy or other medical comorbidities emerging well before an ASD diagnosis can be made. This early identification opens the door for preemptive interventions that might change developmental trajectories and improve outcomes. However, studies are challenged by the wide geographic distribution of families and the presence of medical comorbidities that may preclude travel to clinical sites. These challenges can limit the sample size and generalizability of the cohorts included in both natural history studies and clinical trials [2, 3]. More scalable methods to disseminate research remotely to individuals with rare disorders are urgently needed. Once developed, these methods can translate to other populations that lack adequate access, such as families with limited financial or clinical resources [4–6].

Here, we describe our experience with barriers to access during an ongoing clinical trial for behavioral intervention in infants with Tuberous Sclerosis Complex and the subsequent strategies formulated to mitigate the impact of these barriers. Tuberous sclerosis complex (TSC) is a rare autosomal dominant disorder caused by mutations in the TSC1 or TSC2 gene, occurring in 1 in 7000–13,000 children [7]. TSC is highly penetrant for ASD, with diagnostic rates approaching 60% (compared to up to 2% in the general population) [8–10]. Moreover, because TSC is often diagnosed in utero [11, 12], these infants can be monitored for signs of atypical development well before a clinical ASD diagnosis is made. In a previous longitudinal study of early development, we found that infants with TSC who developed ASD demonstrated marked social communication and nonverbal cognitive delays by age 12 months [13, 14], with deficits in nonverbal communication skills such as eye contact, coordination of gaze, engagement, and social referencing. Despite these clear behavioral markers of atypical development in early infancy, most infants in this study were not receiving targeted social communication interventions, with therapeutic effort focused instead on global development (such as physical therapy to address delayed motor skills).

These natural history study findings prompted the design of a clinical trial of early behavioral intervention targeting social communication skills, with the ultimate goal of improving developmental outcomes. To target the specific nonverbal communicative delays identified in our longitudinal study, a behavioral intervention known as JASPER (Joint Attention, Symbolic Play, Engagement and Regulation) was selected. JASPER has been rigorously studied through clinical trials and has been shown to improve social communication and language skills in toddlers showing red flags for ASD [15]. The clinical trial, called JETS (JASPER Early Intervention in Tuberous Sclerosis, NCT03422367), fills a critical gap in treatment studies in TSC and is the first randomized clinical trial of early behavioral intervention for this syndrome.

**Fig. 1:**
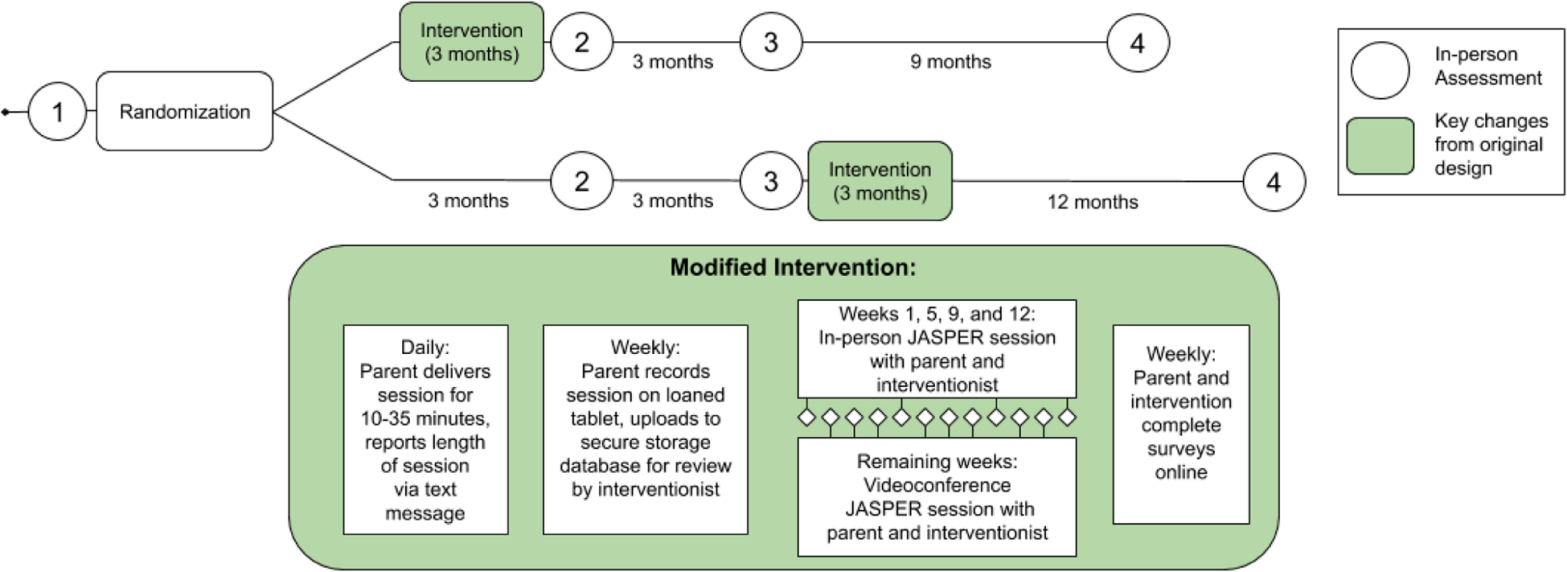
Modified intervention design

There were high expectations that the target enrollment of 60 infants across two study sites would be readily achieved. The initial study included a waitlist-control design (see Fig. 1), with 12 weekly in-person visits over a 3-month active intervention period to one of the two study sites (Los Angeles and Boston) for parent-education based behavioral intervention. Comprehensive in-person assessments were to be performed across four additional time points: pre-intervention, post-intervention, 6-month follow-up, and a 1-year follow-up. However, after one full year of the study, despite active national recruitment efforts supported by the TSC patient alliance group (Tuberous Sclerosis Alliance) and local TSC clinics, only 3 infants were enrolled.

In response to this recruitment challenge, a two-phase process was undertaken to (1) identify the barriers faced by interested families who decided not to enroll and then to (2) make modifications to the study design based upon these findings. Data from parent interviews on barriers to enrollment (phase 1) informed changes made to the research design to improve access (phase 2). Changes in enrollment rate and parent perception following the study design modifications motivate a discussion about the implications of this type of remote delivery for improved access in clinical trials across neurodevelopmental disorders.

## Phase 1: barrier identification

### Methods

#### Recruitment

Recruitment of infants with TSC between 12 and 36 months for the clinical trial was attempted through a variety of well-established mechanisms: TSC specialty clinics, National TS Alliance referrals, online social media postings, and institution-specific medical record queries. Eligible infants had a clinical diagnosis of TSC, with the only exclusion being a planned epilepsy surgery during the trial period.

#### Screening interview

All caregivers who responded to an advertisement or physician/alliance referral (*n* = 25) were contacted via phone. To screen infants for eligibility, caregivers were asked about the age of the child, their clinical diagnosis of TSC, and any upcoming epilepsy surgeries. All 25 of the contacted participants screened as eligible. Staff explained study details and answered questions about participation. Caregivers were then asked whether they would like to enroll in the study, either immediately following screening or after several days if parents requested time to consider.

#### Barriers to enrollment

Twenty-two of 25 caregivers chose not to enroll following the informational screening. These 22 families were asked two open-ended questions regarding their decision: (1) “What were the factors that led to your decision to not enroll in the study?” (2) “Are you interested in future contact if other enrollment options become available?” The results from these interviews were transcribed and categorized.

#### Collection of demographics

Caregivers who enrolled in the study completed a demographics questionnaire at entry, providing information about income, race and ethnicity, and education level.

### Results

#### Barrier identification

**Fig. 2:**
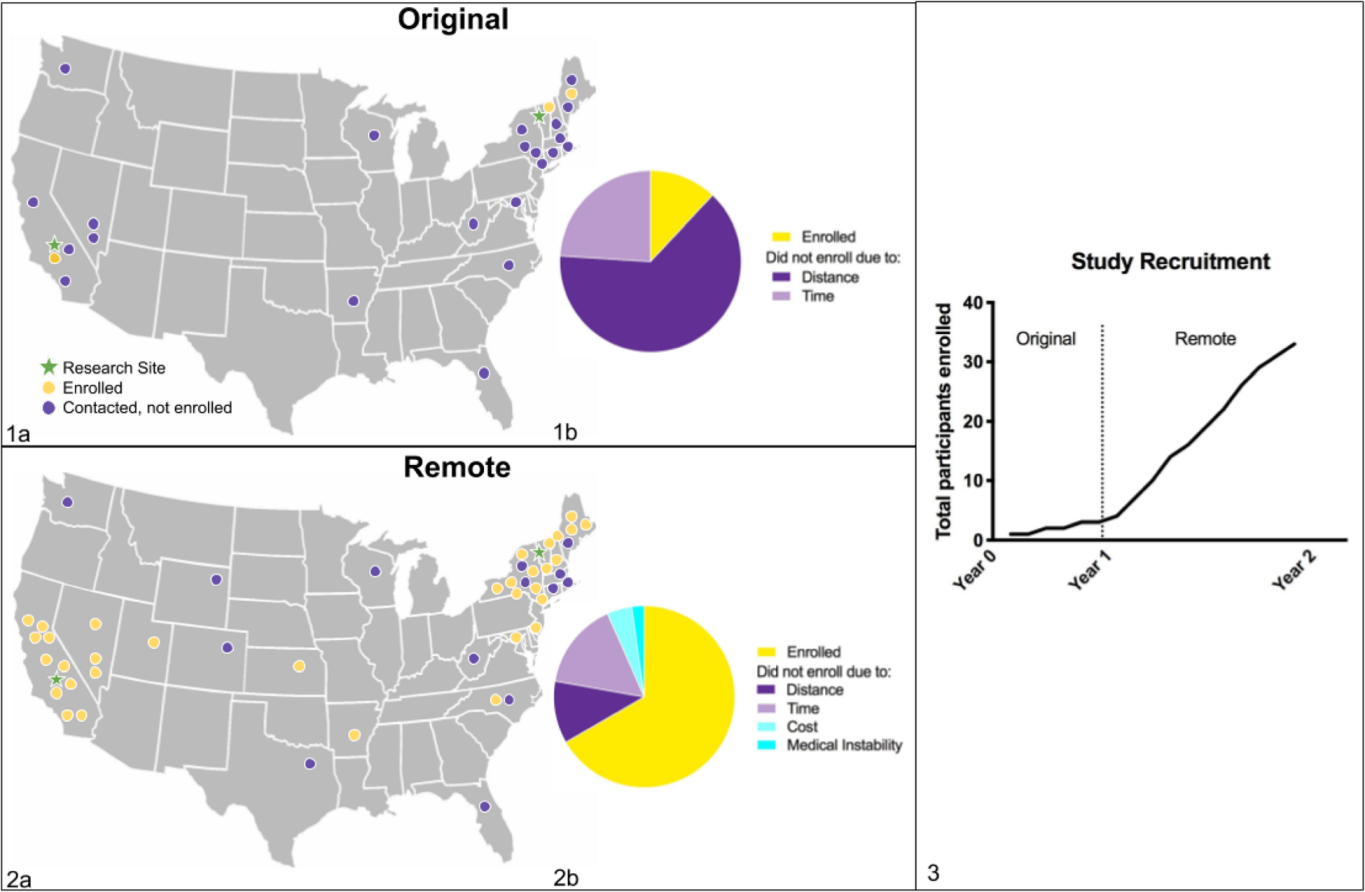
Map of participants with enrollment status and breakdown of enrollment barriers for original design (1a and 1b) and remote design (2a and 2b); Total number of participants enrolled in study with original and remote design (3)

The enrollment yield for the in-person trial design was 12%. Interviews with parents who did not enroll yielded responses that fell into one of two categories, both indicating logistical challenges. The first barrier was time (27%), which included responses related to work schedules, availability of a secondary caregiver, and frequency of appointments; the second barrier was distance (72%) which included responses related to concern for travel costs and flying with their child (see Fig. 2). None of the responses indicated a lack of study buy-in or perceived importance of the research. All families agreed to be recontacted and many expressed a desire to participate under alternate circumstances. For example, one caregiver stated “We are extremely disappointed and hope that another trial will come up for my little one,” and another commented, “If virtual ever becomes an option, we’d be interested.”

## Phase 2: addressing barriers

### Methods

#### Overview of remote caregiver training model

After identifying consistent challenges in recruiting families for participation in weekly in-person intervention sessions, a remote delivery method of intervention was developed to decrease demands of time and travel. The new model (Fig. 1) represents an adaptation of an existing protocol being used by Kasari et al. to remotely train JASPER interventionists nationally and internationally, with the modifications made to train caregivers [15–18]. The protocol includes teaching intervention skills and presenting curriculum content by video conference, asking trainees to practice their newly acquired techniques and to record one of the practice sessions, and submitting videos for review by a training team at one of the study sites [18]. All weekly questionnaires were digitalized. To accommodate this model for caregiver training, the study team consulted with the Security Compliance Office to identify secure and HIPAA-compliant platforms for intervention delivery, resulting in an Institutional Review Board (IRB) approved protocol. The original study design required weekly travel for 100% of sessions, while in the modified design, 70% of sessions are completed remotely. This change reduced the number of in-person hours (assessments, live intervention sessions, and questionnaires) from 33 to 19 and the total number of participation hours (including video conference sessions and online questionnaires) from 39 to 30. Financial reimbursement was moderately increased from $40 to $140, with one night of lodging provided to families at a hotel near the study sites. Families were referred to private funding sources such as National Organization for Rare Disorders (NORD) for further reimbursement.

#### Detailed protocol of caregiver training model

Under the new model, caregivers first attended an in-clinic intervention session focused on active coaching, live demonstration, and direct feedback. Caregivers then received an encrypted tablet to use for the duration of the intervention, with detailed operation instructions and a live demonstration. Caregivers returned home with the goal of practicing JASPER strategies daily. An automated text messaging tool allowed caregivers to easily report their intervention practice time on a daily basis by responding to nightly text. Once per week, caregivers recorded a 30-min practice video on the tablet and uploaded it to a private and secure server. After the weekly upload, a trained interventionist at one of the study sites reviewed the video. Then, during a 30–60 min weekly video conference with the caregiver, the JASPER interventionist provided feedback and introduced new JASPER content for the following week. Content built weekly to introduce new strategies and increase complexity (based on intervention presented in [16]). Weeks 1, 5, 9, and 12 (4 total) took place in-clinic; weeks 2, 3, 4, 6, 7, 8, 10, and 11 (8 total) took place remotely. To collect data on parent fidelity and perspectives, caregivers and interventionists completed weekly questionnaires online through an internal institutional database. Two of these sessions (1 and 12) were combined with visits to study sites for in-person assessments to further reduce travel and scheduling burden on families.

Following these modifications, all 22 previously interested families were recontacted with the option to enroll in the remote intervention model. New families were also contacted for enrollment. Caregivers who chose not to enroll were administered the qualitative interview regarding their decision.

### Results

Six months after the modification to remote delivery, half of the recontacted participants (11/22) enrolled in the study, with the remaining caregivers continuing to cite distance (45%) or time (54%) as barriers to enrollment. Within 1 year of the intervention modification, 23 new families expressed interest, resulting in the enrollment yield increasing to 82% for newly contacted participants (19/23). The four new caregivers who expressed interest in the study but were ultimately unable to enroll identified their primary barrier for enrollment as either time (25%), distance (50%), or medical instability due to epilepsy (25%) as the primary barrier.

Although the sample size of the initial cohort was very small (*n* = 3) compared to the expanded cohort (*n* = 30), a comparison of the two cohorts for descriptive purposes was performed, indicating that the new cohort included a more diverse and representative sample of participants. The percentage of families earning < $90,000 per year increased from 0 to 23%, the percentage of families identified as non-white increased from 0 to 33%, and the percentage of parents without a college degree increased from 0 to 27%. Additionally, the average distance of the participants from the research site increased from 42 to 320 miles, demonstrating the improved geographical reach attained by the modified remote intervention. Following enrollment in the study, a caregiver stated “We live in a rural area where no one has heard of TSC, so it is great to be able to talk to the experts and know they have an eye on my child! I love being able to learn and add to my parent toolbox from my own home, and know I can practice these skills with her every day,” while another said, “We decided when we got the diagnosis that we would do anything and go anywhere to help our son, but doing the intervention at home has been made our participation so much more manageable. I tell every parent I meet that this is a must-do.”

## Discussion

Here, we describe a rapid acceleration in clinical trial enrollment accomplished by the deployment of a remote intervention and assessment strategy. This modification of trial design was necessitated by barriers to access that precluded initial patient enrollment of an already funded clinical trial. Although we did not set out to systematically compare different strategies for enhanced enrollment, the narrative provided here introduces critical themes around enrollment barriers and remote delivery methods that are not unique to TSC. There is a high unmet need for treatment studies in rare neurodevelopmental disorders, yet challenges raised by the geographical distribution and the complex medical needs of these individuals often hamper effective recruitment and study success. The cost to participate in research, particularly intervention, is not only financial (lodging, airfare, meals) but also psychosocial, with disruption of routines and schedules, absence from home and work, and added caregiver burden. Study participation often requires caregivers to commit time during weekdays, requiring flexibility of employment hours, additional childcare, and availability of secondary caregivers. Other clinical trials for rare neurodevelopmental disorders have also begun to use remote intervention strategies, including the NeuroNext trial for Fragile X Syndrome (NCT02920892), which provides language intervention in conjunction with pharmacotherapy through a combination of clinic visits and at-home synchronous video conferencing sessions. Telehealth has additionally benefitted clinical care through models such as a hub and spoke network delivery system in which an anchor “hub” provides comprehensive expertise to secondary “spokes”, including local healthcare providers [19]. One such model known as the Extension for Community Healthcare Outcomes (ECHO) has been applied to Phelan-McDermid Syndrome [20] and provides a platform for video-consultation between local physicians and academic experts to support clinical care in communities, thereby reducing the burden of care on both healthcare providers and caregivers. These telehealth models may prove effective in addressing challenges in recruitment for clinical trials, and importantly, disseminating academic expertise and evidence-based intervention to communities.

There are certainly limitations to remote delivery, including the potential for compromised standardization of protocols, reduced commitment to participation due to the lack of personal contact with study staff, and variable access to and comfort with technology for caregivers. More technological sophistication could further streamline and simplify the process for families, such as the use of a single cell phone application for data collection and intervention, or the replacement of all in-person visits with remote assessment tools. Varying doses of remote delivery need to be tested to determine the minimum amount of live interaction required to achieve measurable outcomes of interest.

Our remote adaptation of intervention directly addresses the barrier of distance, thus greatly increasing enrollment in the study from a geographically diverse pool of participants. Descriptively, the remote intervention cohort is also more representative than the small in-person cohort (a higher percentage of non-white and non-college educated caregivers); however, due to funding restrictions, the financial burden of travel was not fully addressed, and this barrier continues to limit the socioeconomic diversity of participants. In addition, there may be an inherent recruitment bias in the families that chose to engage in remote intervention, possibly selecting for families of infants that are more severely affected by comorbidities, lack financial resources, have limited access to clinically recommended early intervention programs, or live farther from TSC centers of excellence. One might argue that the subset of families who chose to enroll may not represent the broader TSC patient population due to selection factors, thus influencing the generalizability of this type of remote intervention delivery. To address this possible bias, we might consider the subgroup of families who were initially contacted for in-person intervention and then recontacted after modification. Of these families, 10% enrolled in the in-person intervention and 50% elected to participate when a remote option was made available, a lower proportion than the subsequent 82% that enrolled upon first contact. We did not collect demographic or clinical information on the infants that did not enroll, but this distinction does suggest inherent differences in cohorts based on study design and delivery methods that need to be considered when clinical outcomes are determined. 

This study represents an important effort to increase access to intervention clinical trials, which will continue to remain the fundamental obstacle in treatment of rare neurodevelopmental disorders. The paradigm must shift from considering the “gold standard” as research conducted in academic centers to studies that promote family-centered, home-based delivery that maximize participation of all affected children. We also must emphasize that the data presented here are descriptive and meant to generate discussion around the need for innovation in trial design and delivery to maximize access and participation in rare neurodevelopmental disorders. From this experience, we may not be able to conclude that this particular remote delivery strategy is more effective for recruitment than alternative methods, such as the provision of in-home sessions with interventionists, web-based information delivery, or direct recruitment and intervention delivery that coincide with clinic appointments. A critical future direction will be to directly compare enrollment achieved through these various strategies.

## Conclusion

Modification of this clinical trial to include remote delivery of intervention enhanced enrollment tenfold within 1 year and greatly improved the geographic and socioeconomic reach of the study. The striking, rapid surge in enrollment with these adjustments reinforces the tremendous motivation of these families to participate in clinical trials, as well as the need for behavioral intervention and rigorous testing of new approaches to enhance access to research in rare neurodevelopmental disorders. This telehealth model can facilitate future studies not only in rare conditions, but also in other populations that lack adequate access, such as families with limited financial or clinical resources. This study represents an important step towards building an evidence base for remote strategies to promote scalable access to intervention for children with rare genetic disorders, and it reveals a critical direction for future research inquiry as we identify which delivery methods are most effective for diverse populations.

## Data Availability

The datasets generated and/or analyzed during the current study will be available in the NDAR repository upon completion of study.

## Abbreviations

ASD: Autism spectrum disorder
ECHO: Extension for Community Healthcare Outcomes
IRB: Institutional Review Board
JASPER: Joint Attention, Symbolic Play, Engagement and Regulation
JETS: JASPER Early Intervention for Tuberous Sclerosis
TSC: Tuberous sclerosis complex
